# Scalable, methanol‐free manufacturing of the SARS‐CoV‐2 receptor‐binding domain in engineered *Komagataella phaffii*

**DOI:** 10.1002/bit.27979

**Published:** 2021-11-15

**Authors:** Neil C. Dalvie, Andrew M. Biedermann, Sergio A. Rodriguez‐Aponte, Christopher A. Naranjo, Harish D. Rao, Meghraj P. Rajurkar, Rakesh R. Lothe, Umesh S. Shaligram, Ryan S. Johnston, Laura E. Crowell, Seraphin Castelino, Mary K. Tracey, Charles A. Whittaker, J. Christopher Love

**Affiliations:** ^1^ Department of Chemical Engineering Massachusetts Institute of Technology Cambridge Massachusetts USA; ^2^ Koch Institute for Integrative Cancer Research Massachusetts Institute of Technology Cambridge Massachusetts USA; ^3^ Department of Biological Engineering Massachusetts Institute of Technology Cambridge Massachusetts USA; ^4^ Serum Institute of India Pvt. Ltd. Pune India

**Keywords:** COVID‐19, genetic engineering, microbial engineering, *Pichia pastoris*, recombinant protein, subunit vaccine

## Abstract

Prevention of COVID‐19 on a global scale will require the continued development of high‐volume, low‐cost platforms for the manufacturing of vaccines to supply ongoing demand. Vaccine candidates based on recombinant protein subunits remain important because they can be manufactured at low costs in existing large‐scale production facilities that use microbial hosts like *Komagataella phaffii* (*Pichia pastoris*). Here, we report an improved and scalable manufacturing approach for the SARS‐CoV‐2 spike protein receptor‐binding domain (RBD); this protein is a key antigen for several reported vaccine candidates. We genetically engineered a manufacturing strain of *K. phaffii* to obviate the requirement for methanol induction of the recombinant gene. Methanol‐free production improved the secreted titer of the RBD protein by >5X by alleviating protein folding stress. Removal of methanol from the production process enabled to scale up to a 1200 L pre‐existing production facility. This engineered strain is now used to produce an RBD‐based vaccine antigen that is currently in clinical trials and could be used to produce other variants of RBD as needed for future vaccines.

## Graphical abstract

The authors report an improved low‐cost manufacturing process in yeast for a component of a clinical‐stage COVID‐19 vaccine candidate. An engineered strain of Pichia pastoris produces the antigen without using methanol, improving the secreted protein titer and cell health. Elimination of methanol as a process requirement enabled rapid technology transfer to an existing large‐scale production facility.

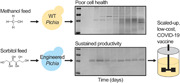

## 1 INTRODUCTION

As new variants of severe acute respiratory syndrome coronavirus 2 (SARS‐CoV‐2) emerge, continued development of diagnostics, vaccines, and reagents remains essential to address the COVID‐19 pandemic. The SARS‐CoV‐2 spike protein is an essential reagent for serological assays, and a component of several protein‐based vaccines (Guebre‐Xabier et al., 2020; Tian et al., 2021). Vaccine candidates based on protein subunits are also important ones for enabling interventions for the pandemic in low‐ and middle‐income countries due to existing large‐scale manufacturing facilities and less stringent temperature and storage requirements for distribution (Dai et al., 2020). We and others have reported vaccine designs based on the receptor‐binding domain (RBD) of the spike protein (Dalvie et al., 2021). In these designs, the RBD can be produced independently, and subsequently, displayed on protein or lipid nanoparticles for enhanced immunogenicity (Cohen et al., 2021; Walls et al., 2020). The 201 amino acid RBD is an especially promising antigen for accessible vaccines because it can be manufactured at low cost and with high volumes in microbial hosts (Chen et al., 2020; Pollet et al., 2021). Here, we report an engineered yeast strain with enhanced secretion of the SARS‐CoV‐2 RBD from the circulating variants of Wuhan Hu‐1, B.1.1.7, and B.1.351 strains of the virus. This engineered host has been successfully deployed at a 1200 L scale to produce a vaccine component currently in clinical trials.

The methylotrophic yeast *Komagataella phaffii* (*Pichia pastoris*) is routinely used for the production of therapeutic proteins at large volumes because of its high‐capacity eukaryotic secretory pathway (K. R. Love et al., 2018). Another key advantage of this production host is the strong, tightly regulated, methanol‐inducible promoter, P_AOX1_, used for the expression of the recombinant gene (Ahmad et al., 2014). This promoter enables outgrowth to high cell densities with inexpensive feedstock like glycerol before induction of the recombinant gene with methanol feed. Methanol can pose challenges, however, in large‐scale facilities, including high heat generation during fermentation and flammability concerns while in storage (Potvin et al., 2012). The impact of these challenges is that facilities require specific designs or modifications to handle methanol. This requirement could limit the number of manufacturing facilities available for the production of vaccine components like the RBD antigens in *K. phaffii* in a pandemic. We sought to reduce or eliminate the requirement for methanol for efficient secretion of the RBD.

**Figure 1:**
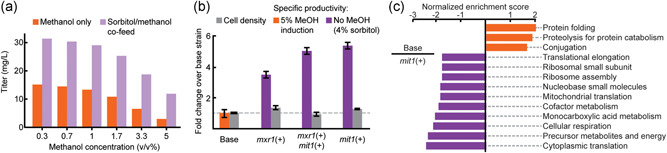
Improved productivity and decreased stress in methanol‐free RBD expression. (a) Approximate titers of secreted RBD from individual cultures of the base strain in 3‐ml plate culture, measured by reverse‐phase liquid chromatography. (b) Performance of three engineered strains in 3‐ml plate culture. Error bars represent the standard deviation of three biological replicates. (c) Enriched gene sets between the base strain (orange) and the *mit1+* strain (purple). RBD, receptor‐binding domain; v/v, volume per volume

We previously reported the production of the SARS‐CoV‐2 RBD (Wuhan‐Hu‐1 sequence) in an engineered variant of *K. phaffii* (Brady et al., 2020; Dalvie et al., 2021). To assess the feasibility of methanol‐free production, we cultivated the strain expressing RBD regulated under the native AOX1 promoter, and induced expression of the recombinant gene with varying amounts of methanol (Figure 1a). Interestingly, the approximate secreted titers of RBD increased as the concentrations of methanol were reduced. We also induced protein production with a combination of methanol and sorbitol—a supplementary carbon source that does not repress P_AOX1_ expression—and observed a further increase in titer.

Given our observation of improved productivity with reduced quantities of methanol in batch cultivations, we hypothesized that we could achieve or maintain productive secretion of the RBD in the absence of methanol with suitable engineering of the strain. The expression of genes regulated by P_AOX1_ in wild‐type *K. phaffii* in the absence of methanol is inconsistent, even with nonrepressive carbon sources like sorbitol (Vogl et al., 2018). Several studies, however, have demonstrated that constitutive overexpression of activating transcription factors like *mit1* and *mxr1* can lead to consistent activation of P_AOX1_ without methanol (Shi et al., 2019; Vogl et al., 2018). To test the production of RBD without methanol, we integrated additional copies of the endogenous transcription factors *mit1* and *mxr1* into the *K. phaffii* genome under a glycerol‐repressible promoter (Dalvie et al., 2019). We cultivated these strains for protein production by feeding with only sorbitol (Figure 1b). We observed a >threefold increase in specific productivity in all strains, particularly with a strain containing only one extra copy of the transcription factor *mit1* (>fivefold).

To assess the potential source of improved productivity, we performed a comparison of the methanol‐fed base strain and the modified, sorbitol‐fed *mit1*+ strain. We observed no intracellular accumulation of RBD protein in either strain (Figure S1a). Next, we examined the transcriptomes of the methanol‐fed initial strain and the modified, sorbitol‐fed *mit1*+ strain by RNA‐sequencing. The sorbitol‐fed *mit1*+ strain appeared to produce less RBD transcript than the methanol‐fed base strain, but the difference was not significant (unpaired *t*‐test, *p* = 0.06) (Figure S1b). We analyzed the variations in gene expression by gene set enrichment analysis (GSEA; Figure 1c). We observed significantly higher expression of genes associated with protein folding stress in the methanol‐fed condition compared to the sorbitol‐fed *mit1*+ condition (family‐wise error *p* = 0.003). These results suggest that sorbitol‐fed *mit1*+ may improve productivity by mitigating protein folding stress associated with RBD production.

To determine whether the observed reduction in protein folding stress was due to the sorbitol feed or the *mit1*+ engineering, we cultivated the *mit1*+ strain with different feed conditions (Figure S1c). We observed that the specific productivity of secreted RBD was reduced in 5% methanol feed, even with the *mit1*+ engineering. The improvement in specific productivity, therefore, can be primarily attributed to the elimination of methanol as a carbon source. This observation is consistent with previous transcriptomic studies about methanol metabolism in *K. phaffii* (Lin et al., 2021; Vanz et al., 2012). Further studies are warranted to determine the interplay between the transcript level and types and quantities of carbon source on productivity.

**Figure 2:**
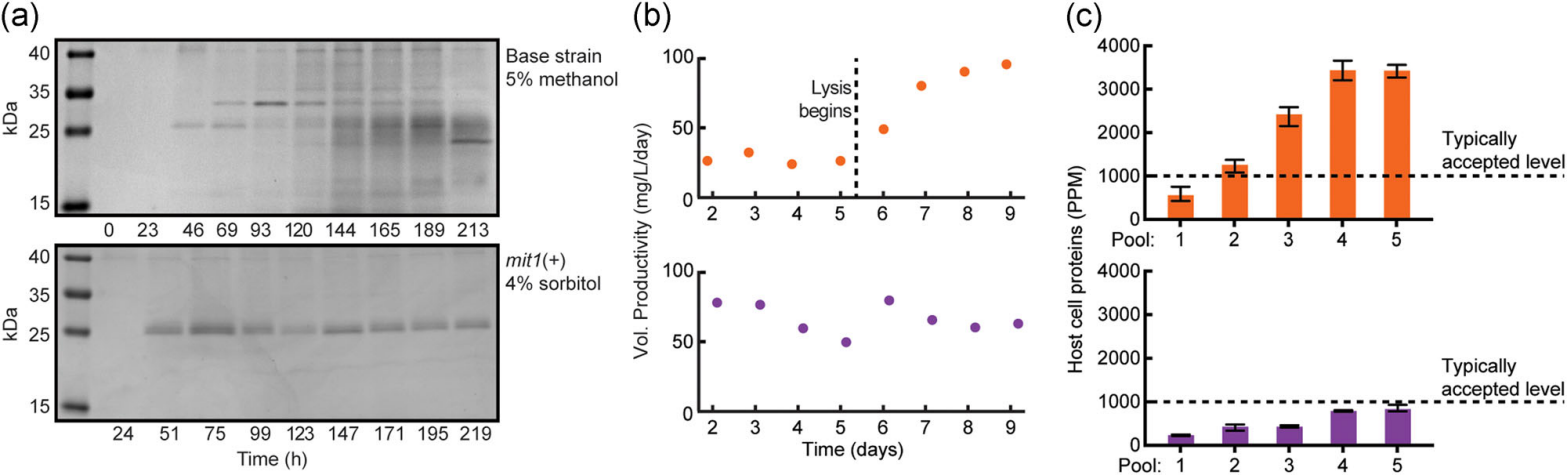
Sustained productivity of the methanol‐free strain in perfusion fermentation. (a) Reduced SDS‐PAGE of upstream reactor samples for the duration of each campaign. (b) Upstream reactor titer of RBD. (c) Host cell protein concentrations in purified pools of RBD, measured by ELISA. Error bars represent the standard deviation of three technical replicates. ELISA, enzyme‐linked immunoassay; PPM, parts per million; RBD, receptor‐binding domain; SDS‐PAGE, sodium dodecyl sulphate–polyacrylamide gel electrophoresis

After comparing the specific productivity of the methanol‐free strain (*mit1*+) to the methanol‐induced (base) strain, we assessed the production of RBD using both strains on InSCyT, a continuous, automated, perfusion‐based manufacturing platform (Crowell et al., 2018). The base strain exhibited low titers (∼30 mg/L) in perfusates and significant cell lysis after ∼120 h of fermentation in perfusion (Figure 2a,b). In contrast, the *mit1*+ strain maintained protein secretion at >50 mg/L/day for the duration of a >200 h campaign. RBD purified from the perfusates produced by the base strain also contained more host‐related impurities than RBD from the *mit1*+ campaign (Figure 2c). These results from the sustained production of RBD, including the cell lysis observed in the base strain, are consistent with the observations for increased cellular stress relative to the *mit1*+ strain, and suggest the transcriptional changes observed also translated into variation in protein expression as well.

**Figure 3:**
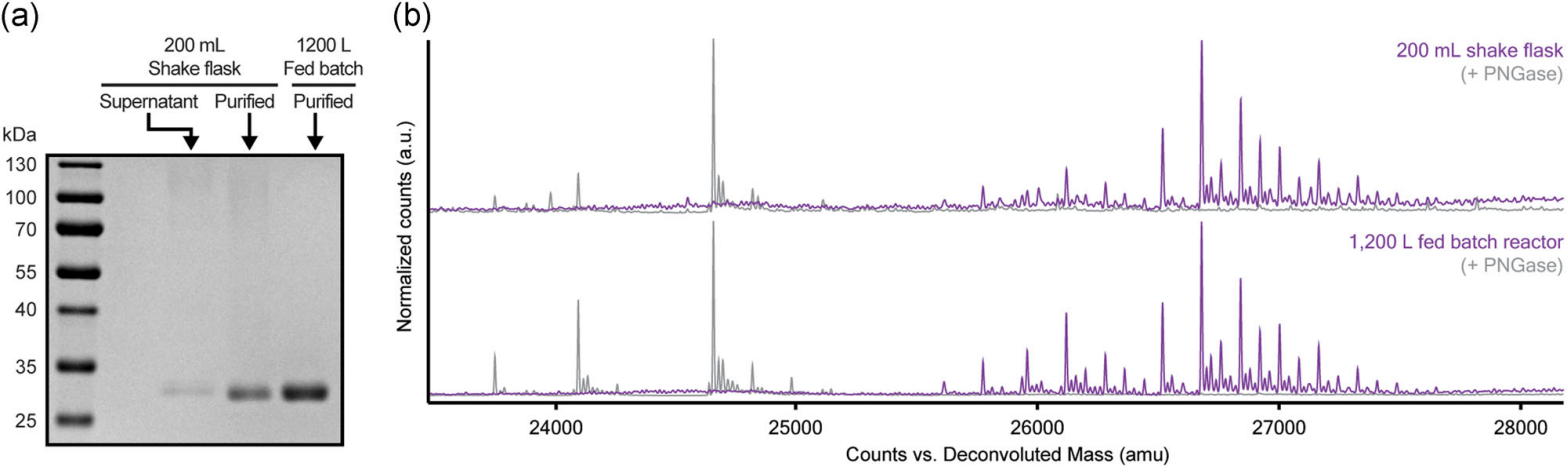
RBD‐SpyTag produced at lab scale and GMP scale. (a) Reduced SDS‐PAGE of RBD‐SpyTag in crude shake flask supernatant, purified from shake flask cultivation, and purified from a fed‐batch process. (b) Intact mass spectra of purified RBD‐SpyTag from each manufacturing process. Overlayed spectra are before and after treatment with PNGase. GMP, good manufacturing practice; RBD, receptor‐binding domain; SDS‐PAGE, sodium dodecyl sulphate–polyacrylamide gel electrophoresis

From these data for the improved production of RBD in bioreactors with the modified strain without methanol, we then generated a *mit1*+ strain that expressed RBD with a C‐terminal fusion of SpyTag, a short peptide that can mediate a transpeptidation reaction with a cognate SpyCatcher polypeptide, which can be presented on protein nanoparticles for example (Reddington & Howarth, 2015). We expressed and purified the RBD‐Spytag from this strain in a 200 ml shake flask culture. We also transferred this *mit1*+ strain encoding RBD‐SpyTag, to a facility for good manufacturing practice manufacturing in a 1200‐L fed‐batch process. In this process, the strain produced 21 mg per liter of fermentation of purified, clinical quality RBD‐SpyTag, or approximately >1 million doses from a single reactor batch, assuming a vaccine formulation with 25 µg of RBD‐SpyTag per dose. The two purified products from each production scale were similar by sodium dodecyl sulphate–polyacrylamide gel electrophoresis (SDS‐PAGE), and exhibit nearly identical glycan profiles, indicating consistency in the quality attributes of the molecules produced at these two scales with this modified strain for methanol‐free production (Figure 3).

**Figure 4:**
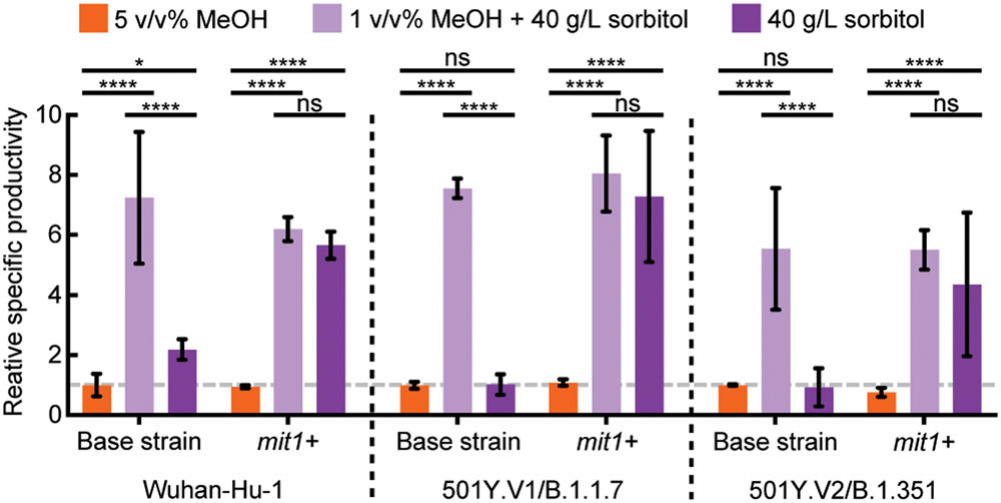
Methanol‐free production of RBD variants in 3‐ml culture. Error bars represent standard deviation across three biological replicates. Significance was determined by multiple *t*‐tests with Holm Sidak correction. *ns*, not significant; RBD, receptor‐binding domain. *p < 0.01, ****p < 0.000001

We next sought to assess whether or not this modified *mit1*+ strain could improve the production of sequence variants for other circulating SARS‐CoV‐2 virus strains as well. We generated strains expressing RBD‐B.1.1.7 and RBD‐B.1.351 in both the base and *mit1*+ strain backgrounds, and evaluated their specific productivities in different media for production (Figure 4). In all strains, reduced methanol feed improved productivity. For all RBD variants, only *mit1*+ engineered strains maintained improved productivity in the absence of methanol. This result demonstrates that the engineered *mit1*+ strain could facilitate new cell lines for manufacturing other RBD variants without methanol for seasonal vaccine boosters or next‐generation vaccine candidates for emerging variants.

In conclusion, we report here a strain that enables the manufacturing of SARS‐CoV‐2 RBD variants without methanol. This strain exhibits improved secreted productivity due to a reduction in protein folding stress. We demonstrated sustained productivity from the strain in a perfusion process, and scale‐up to a large‐scale, methanol‐free fed‐batch process to produce a vaccine component currently in clinical trials. In this case, manufacturing at the 1200 L scale was possible with the elimination of the requirement for methanol in the medium. Strains engineered for use without methanol and increased productivity could facilitate the manufacturing of RBD and other antigens for vaccine candidates at large volumes and low costs to enable accessible and affordable vaccines for global use.

## 2 MATERIALS AND METHODS

### 2.1 Yeast strains

All strains were derived from wild‐type *K. phaffii* (NRRL Y‐11430). The base strain was described previously (Brady et al., 2020). The gene containing the RBD was codon‐optimized, synthesized (Integrated DNA Technologies), and cloned into a custom vector. The RBD vector was transformed as described previously (Dalvie et al., 2019). Transcription factors *mit1* and *mxr1* were integrated into the genome near genomic loci GQ67_02967 and GQ67_04576, respectively, using a markerless CRISPR‐Cas9 system described previously (Dalvie et al., 2019). Both *mit1* and *mxr1* were under the control of the P_CAT1_ promoter from *K. phaffii*. Sequences for P_CAT1_, *mit1*, and *mxr1* were amplified from the *K. phaffii* genome. All plasmid sequences are included in the Supporting Information.

### 2.2 Cultivations

Strains for initial characterization and titer measurement were grown in 3 ml culture in 24‐well deep‐well plates (25°C, 600 rpm), and strains for protein purification were grown in 200 ml culture in 1 L shake flasks (25°C, 250 rpm). Cells were cultivated in Rich Defined Media, described previously (Matthews et al., 2018). Cells were inoculated at 0.1 OD600, outgrown for 24 h with 4% glycerol feed, pelleted, and resuspended in fresh media with methanol or sorbitol feed to induce recombinant gene expression. Supernatant samples were collected after 24 h of production, filtered, and analyzed. InSCyT bioreactors and purification modules were operated as described previously (Crowell et al., 2018; Dalvie et al., 2021).

### 2.3 Analytical assays for protein characterization

Purified protein concentrations were determined by absorbance at A280 nm. SDS‐PAGE was carried out as described previously (Crowell et al., 2018). Supernatant titers were measured by reverse‐phase liquid chromatography (LC) as described previously (Dalvie et al., 2021), and normalized by cell density, measured by OD600. Intact mass spectrometry was performed as described previously (Dalvie et al., 2021) but with the following modifications: LC gradient of 5%–95% solvent B over 4 min at a flow rate of 0.8 ml/min, and 250 V fragment or voltage.

### 2.4 Transcriptome analysis

Cells were harvested after 18 h of production at a 3 ml plate scale. RNA was extracted and purified according to the Qiagen RNeasy Kit (cat #74104) and RNA quality was analyzed to ensure RNA quality number >6.5. RNA libraries were prepared using the 3′‐digital gene expression method and sequenced on an Illumina Miseq to generate paired reads of 20 (Read 1) and 72 bp (Read 2). Sequenced messenger RNA transcripts were demultiplexed using sample barcodes and polymerase chain reaction duplicates were removed by selecting one sequence read per unique molecular identifier using a custom python script. Transcripts were quantified with Salmon version 1.1.0 (Patro et al., 2017) and selective alignment using a target consisting of the *K. phaffii* transcripts, the RBD, and selectable marker transgene sequences, and the *K. Phaffii* genome as a selective alignment decoy. Expression values were summarized with tximport version 1.12.3 (Soneson et al., 2016) and edgeR version 3.26.8 (McCarthy et al., 2012; Robinson et al., 2009). Expression was visualized using *log*_2_ *(Counts per Million *+ *1)* values. GSEA was performed with GSEA 4.1.0 using Wald statistics calculated by DESeq. 2 (M. I. Love et al., 2014) and gene sets from yeast gene ontology slim (Subramanian et al., 2005).

## CONFLICT OF INTERESTS

Laura E. Crowell and J. C. Love have filed patents related to the InSCyT system and methods. Laura E. Crowell is a current employee at Sunflower Therapeutics PBC. J. C. Love has interests in Sunflower Therapeutics PBC, Pfizer, Honeycomb Biotechnologies, OneCyte Biotechnologies, QuantumCyte, Amgen, and Repligen. J. C. Love's interests are reviewed and managed under MIT's policies for potential conflicts of interest. Harish D. Rao, Meghraj P. Rajurkar, Rakesh R. Lothe, and Umesh S. Shaligram are employees of Serum Institute of India Pvt. Ltd. Other authors have no conflict of interests.

## AUTHOR CONTRIBUTIONS

Neil C. Dalvie, Andrew M. Biedermann, and J. Christopher Love conceived and planned experiments. Andrew M. Biedermann and Neil C. Dalvie designed and implemented clustered regularly interspaced short palindromic repeats‐based genome modifications. Neil C. Dalvie and Ryan S. Johnston transformed the receptor‐binding domain genes. Andrew M. Biedermann and Charles A. Whittaker performed RNA sequencing. Andrew M. Biedermann conducted plate scale cultivations. Andrew M. Biedermann, Seraphin Castelino, and Mary K. Tracey conducted InSCyT experiments. Sergio A. Rodriguez‐Aponte and Andrew M. Biedermann performed HPLC assays. Sergio A. Rodriguez‐Aponte and Laura E. Crowell designed and performed protein purifications. Christopher A. Naranjo performed mass spectrometry. Harish D. Rao, Meghraj P. Rajurkar, Rakesh R. Lothe, and Umesh S. Shaligram performed and oversaw scale‐up to 1200 L fed‐batch. Neil C. Dalvie, Andrew M. Biedermann, and J. Christopher Love wrote the manuscript. All authors reviewed the manuscript.

## Supporting information

Supporting information.

Supporting information.

## Data Availability

RNA sequencing data is available in the NCBI Gene Expression Omnibus, accession GSE183408.
