# Supplementary material for: Scalable, methanol‐free manufacturing of the SARS‐CoV‐2 receptor‐binding domain in engineered Komagataella phaffii
Source: Biotechnol Bioeng. 2021 Nov 15;119(2):657–62. doi: 10.1002/bit.27979 (PMC8653030; doi:10.1002/bit.27979)
Supplement: Supplementary file 1 — Supporting information. [file BIT-119-657-s001.docx]

**Scalable, methanol-free manufacturing of the SARS-CoV-2 receptor binding domain in engineered *Komagataella phaffii***

Supporting Information

Neil C. Dalvie^1,2^^, Andrew M. Biedermann^1,2^^, Sergio A. Rodriguez-Aponte^2,3^, Christopher A. Naranjo^2^, Harish D. Rao^4^, Meghraj P. Rajurkar^4^, Rakesh R. Lothe^4^, Umesh S. Shaligram^4^, Ryan S. Johnston^2^, Laura E. Crowell^1,2^, Seraphin Castelino^1^, Mary Kate Tracey^2^, Charles A. Whittaker^2^, J. Christopher Love^1,2^*

^1^Department of Chemical Engineering, Massachusetts Institute of Technology, Cambridge, Massachusetts 02139, United States

^2^The Koch Institute for Integrative Cancer Research, Massachusetts Institute of Technology, Cambridge, Massachusetts 01239, United States

^3^Department of Biological Engineering, Massachusetts Institute of Technology, Cambridge, Massachusetts 02139, United States

^4^Serum Institute of India Pvt. Ltd., Pune, India

^Contributed equally

*Correspondence to: [clove@mit.edu](mailto:clove@mit.edu)


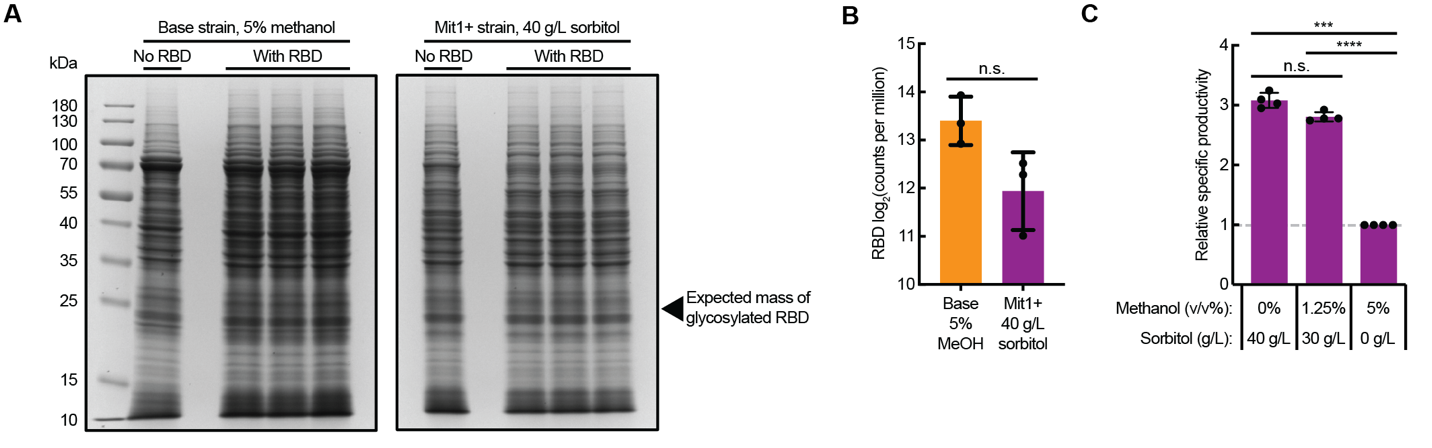


Fig. S1

A) SDS-PAGE of intracellular protein after cultivation of the base strain and the *mit1+* strain, with and without the recombinant RBD gene. The base strain was cultivated with 5% methanol feed, and the *mit1+* strain was cultivated with 40 g/L sorbitol feed. B) Abundance of the recombinant RBD transcript in the base strain and the *mit1+* strain, measured by RNA sequencing. Significance was determined by unpaired t-test. C) Relative specific productivity of the *mit1+* strain cultivated with different feed conditions. Four biological replicates were normalized to the corresponding replicate in the 5% methanol condition. Significance was determined by ratio paired t-test.

****p<0.00001, ***p<0.0001, n.s. indicates p > 0.05

Table S1. Plasmids used in this study

| **Description** | **Locus** | **Integration mechanism** |
| --- | --- | --- |
| Expression vector for RBD | P_AOX1_ | Multi-copy with zeocin selection |
| Expression vector for RBD_B.1.1.7 (Alpha) | P_AOX1_ | Multi-copy with zeocin selection |
| Expression vector for RBD_B.1.351 (Beta) | P_AOX1_ | Multi-copy with zeocin selection |
| Integration vector for MIT1 | GQ67_02967 | Markerless integration with CRISPR |
| Integration vector for MXR1 | GQ67_04576 | Markerless integration with CRISPR |
